# Genome Sequences of Elezi, Asa16, and Niobe, Three Cluster AZ Phages Isolated Using Arthrobacter globiformis *B-2979*

**DOI:** 10.1128/mra.00368-22

**Published:** 2022-08-09

**Authors:** Emanuela Elezi, Erica Sellers, Alia Abdulla, Marisa A. DeCiucis, Brielle R. Lynch, Mark Nowak, Jamie Outlaw, Camila Ramos, Jacklyn Ramos-Arvelo, Sophia Rokas, Yadinitza Torres-Cintrón, Simon Uka, Stefan Uka, Luz D. Vargas, Nicholas P. Edgington

**Affiliations:** a Department of Biology, Southern Connecticut State University, New Haven, CT, USA; Portland State University

## Abstract

The Actinobacteriophages Elezi, Asa16, and Niobe infect Arthrobacter globiformis *B-2979* and are closely related to Eraser and London in Cluster AZ. They have flexible noncontractile tails, are predicted to be temperate phages, and their genome sizes range between 43,471 bp and 43,602 bp.

## ANNOUNCEMENT

Bacteria in the genus *Arthrobacter* are Gram-positive rod-shaped obligate aerobes, and their genomes are considered to have a high GC percent. *Arthrobacter* bacteria exist in the air, water, soil, and some cheeses, and play important roles in the degradation of many different synthetic toxic compounds (1).

Actinobacteriophages Elezi, Asa16, and Niobe were isolated from soil in Connecticut, USA (Table 1) by adding 35 mL of PYCa growth medium to approximately one gram of soil and shaking the samples for 1 h at 250 rpm at 30°C, and then filtering the samples through a 0.22 μm filter. The flowthrough was then inoculated with 0.5 mL of a saturated culture of Arthrobacter globiformis NRRL *B-2979* and incubated at 30°C for 2 days shaking at 250 rpm. All three phages were predicted to be temperate. Consistent with the comparative genomics predictions, transmission electron microscopy of all three phages using 1% uranyl acetate staining of lysates on 200 to 400 mesh carbon-Formvar-coated copper grids showed a siphoviral tail morphology.

**TABLE 1:** Phage genome characteristics and assembly results

Phage	Plaque diam (mm)	GPS coordinates	Genome accession no.	SRA accession no.	Total reads	Fold coverage	Genome size (bp)	GC percent (%)	No. of genes	Percent of hypothetical open reading frames with no known function (%)
Elezi	5	41.554167 N, 72.959722 W	MT639653.1	SRX12198765	421,195	1,377	43,471	66.6	68	41
Asa16	4.74	41.3347 N, 72.9421 W	MZ681506.1	SRX12198764	714,166	2,348	43,601	66.6	69	42
Niobe	8	41.33349 N, 72.945666 W	MZ820087.1	SRX12198770	519,331	1,729	43,602	66.7	69	42

Genomic DNA was isolated from a high-titer lysate of the purified phages using a Promega Wizard DNA Clean-Up kit and prepared for sequencing with an NEB Ultra II v3 reagents Library kit. The samples were sequenced and demultiplexed using an Illumina MiSeq platform with 150-bp single-end reads. A random subset of the total untrimmed reads was assembled, using Newbler v2.9 and Consed v29.0 (2) according to Russell et al. (3) with coverage being between 1,377 and 2,348-fold (Table 1). Based upon an observed buildup of reads in Consed with identical start positions, and rare reads that crossed the ends, the three genomes were predicted to be linear with eleven bases and 3’ single-stranded overhangs with the sequence CGAAGGGGCAT. The phages that infect Arthrobacter globiformis NRRL *B-2979* have been grouped into seventeen ‘Clusters’ and three ‘Singletons’, with the Cluster AZ being the largest cluster of phages that can infect this host (4). These three phages have been assigned to be in Cluster AZ based on sharing 35% or greater of gene content similarity with other Cluster AZ members (5).

Genes were predicted with the programs Glimmer v.3.02b (6) and Genemark v.3.25 (7). A search for tRNA and tmRNA sequences was performed with ARAGORN v.1.2.41 (8) and tRNAscan-SE v.2.09 (9), and none were predicted in any of these three genomes. The genomes were annotated using the web application PECAAN v.20211202 (https://discover.kbrinsgd.org), and DNAMaster v.5.23.6 (10). Average nucleotide identity (ANI) values were calculated for all Cluster AZ phages using PyANI v.0.2.11 (https://github.com/widdowquinn/pyani) (11). Elezi, Asa16, and Niobe were closely related to Eraser and London, with a pairwise ANI average range between 0.9828 and 0.9988. All three genomes have a similar number of total predicted open reading frames (ORFs), and ORFs with no predicted function (Table 1). The genome organization of these three phages is common to many other actinobacteriophages, where the predicted structural genes were on the left side of the linear genome, and more of the ORFs of unknown function reside on the right side of these genomes.

### Data availability.

The complete genome sequences and sequence read archives for Elezi, Asa16, and Niobe are available at NCBI’s GenBank (Table 1).
